# Clinical, diagnostic and Cryptococcosis neuroméningées evolutionary aspects in HIV infection

**DOI:** 10.1186/1742-4690-9-S1-P149

**Published:** 2012-05-25

**Authors:** Rais Mounira, Amel Ouyahia, Abedelkader Gasmi, Wahiba Guenifi, Houda Boukhrissa, Abdelmadjid Lacheheb

**Affiliations:** 1 Maladies Infectieuses at Chu Setif Algerie, Setif, Algeria

## Context

Cryptococcosis is a deep Mycosis of reserved in AIDS prognosis Goal. The objective of our study is to analyze the characteristics of clinical, diagnostic and scalable of the NJC in the PVIH.

## Materials and methods

Descriptive and retrospective study conducted from medical records of positive HIV patients hospitalized for neurological disorders of 2000-2009 in the service of infectious diseases CHU of Sétif.

## Results

In 12 patients hospitalized for neurological disorders, four cases of meningitis, cryptococcal (02 men and 02 women) have been diagnosed. The average age of our patients is 45 years; the HIV contamination was sexual in 100% cases. The NJC was the fact of discovery of the HIV infection in 01 case. All our patients had clinical events ranking stage C, CD4 performed in all patients were < 100/mm^3^. The clinical presentation was variable and the clinical signs most frequently encountered were headache, neck stiffness, the alteration of consciousness, fever and the seizures. Examination of CSF to the ink was contributing to the diagnosis in all cases. The culture of CSF made all our patients was positive. The total lymphocyte count showed lymphopenia with an average number of lymphocytes à 800 /mm^3^. Cases of co-infection were recorded (with Mycobacterium tuberculosis in 1 patient, and pneumocystis jirovecii in another) Amphotericin B (0, 7 mg/kg/day) monotherapy was used in all patients in first intention with relay by fluconazole, or in addition to treatment, or for drug intolerance. The evolution of the NJC has been marked by the death of 04 patients.

